# Characterization of the random positioning machine as a microgravity simulator for biological applications

**DOI:** 10.1371/journal.pone.0351320

**Published:** 2026-06-16

**Authors:** Andrada Pica, Giuseppe Uras, Ilaria Giuseppina Porco, Alessia Manca, Antonella Pantaleo, Ugo Della Croce

**Affiliations:** 1 Department of Engineering, University of Sassari, Sassari, Italy; 2 Department of Biomedical Sciences, University of Sassari, Sassari, Italy; American University of Madaba, JORDAN

## Abstract

Simulated microgravity platforms provide essential tools for studying gravitational effects on biological systems under controlled laboratory conditions. The Random Positioning Machine (RPM) is among the most commonly used ground-based simulators, yet quantitative evaluations of its mechanical performance and biological effects remain limited. This study provides a comprehensive mechanical and biological characterization of an RPM device capable of operating in randomized, unidirectional, and single-axis clinostat modes. Angular velocity profiles were experimentally recorded using magneto-inertial measurement units mounted on the RPM frames. These data informed a computational model that simulated gravity vector dispersion and centrifugal acceleration across different operational configurations. Additionally, SH-SY5Y neuronal cells were exposed to simulated microgravity under each mode to evaluate cellular responses. The computational analysis demonstrated that all RPM modes effectively achieved simulated microgravity conditions, with time-averaged gravity values ranging from 10^−2^ to 10^−3^ g. Centrifugal accelerations remained below 0.08 g across all conditions. Biologically, SH-SY5Y cells exposed to simulated microgravity exhibited reduced confluency and increased α-synuclein inclusions in all RPM configurations, with milder effects observed in clinostat mode. The integration of experimental, computational, and biological analyses establishes a quantitative framework for assessing and optimizing RPM-based microgravity simulations. The findings confirm that both RPM and clinostat modes can reproduce key features of microgravity, while highlighting the role of motion characteristics in shaping biological responses. The proposed computational model represents a predictive tool to support the design and reproducibility of future ground-based microgravity studies.

## Introduction

Gravity constantly influences organisms on Earth, stimulating numerous vital functions at every level of the organism, from molecules to the entire body [1,2]. Extracellular mechanical forces are essential for developmental biology and tissue homeostasis [3,4]. It is well-established that mechanical forces significantly influence human physiology and, similarly, the opposite condition of mechanical unloading also impacts cell functionality and tissue homeostasis [3]. The mechanical unloading effects are observed, for instance, in astronauts exposed to the microgravity (µg) environment during space missions. These effects are diversified, and range from bone demineralization, muscle atrophy, immune system dysfunctions, neurological impairment, to alterations in endocrine and metabolic processes [5–8].

Understanding these physiological changes is crucial for mitigating health risks associated with long-term space missions. Moreover, µg-induced physiological changes, such as loss of muscle mass and decreasing bone density, partially mirror degenerative processes typical of aging. This is one important factor that fostered scientists’ interest in doing space research and undeniably, space biomedicine studies have promoted relevant advancements in medical research in broader contexts [1,6].

However, performing biological experiments in space is costly and logistically complex due to limited space availability, difficulties in delivering experimental instruments, or constraints on astronaut workloads. These limitations have driven the development of ground-based systems to simulate µg on Earth [9–11]. These devices typically simulate gravity levels in the range 10^−2^ to 10^−5^ g. It is important to note that the term “microgravity” does not necessarily imply an environment with gravitational forces of 10^^−6^^ g. Even in space environments, real µg, characterized by accelerations as low as 10^^−6^^ g, is rarely achieved [12]. Instead, gravity levels aboard spacecraft or on the International Space Station typically range between 10^^−3^^ and 10^^−6^^ g [13–15].

Among the most commonly used ground-based simulators are clinostats and random positioning machines (RPMs) which mimic µg conditions by rotating biological specimens such as cell cultures [4,16], small animals [17], and plants [18–21].

The working principle of a 2D clinostat consists in the rotation around one axis perpendicular to the direction of the gravity vector, with a constant angular velocity. This rotation modifies the direction of gravity perceived by the sample over time, with reported residual gravity values typically ranging from 10^^−3^^ g to 10^^−2^^ g. Alternatively, the 3D clinostat uses two perpendicular frames rotating at constant speeds and directions [9,10,22].

A further development in µg simulation technology is represented by the RPM which enables continuous reorientation of the gravity vector in three-dimensional space. The device consists of two nested frames driven by independent motors, enabling samples positioned on the inner frame to rotate around two perpendicular axes. This setup allows for a highly versatile range of movements. Dedicated algorithms drive frame rotation, and the gravity vector is distributed in all directions over time without any directional preference. In the RPM mode, speed and direction are typically different and randomized. Averaged over time, the residual acceleration reaches values between 10^^−3^^ g to 10^^−2^^ g [23–25].

By frequently altering the gravity vector, clinostats and RPM prevent cells from fully adapting, ensuring that the biological response remains in a dynamic state, similar to what is experienced in actual µg [11,26]. Since biological systems require a minimum exposure time to a given gravity vector to adapt, on the order of minutes to hours, this continuous reorientation usually keeps cells in a dynamic, unsettled state [27]. Even though the earth gravity acceleration is always acting on the tested sample, from the sample point of view, the gravitational acceleration vector exhibits a continuously changing orientation that, averaged over time, converges toward zero mathematically [9,10].

Several strategies have been adopted in the design and application of µg analogs, encompassing both the development or refinement of hardware and control algorithms. Different studies have proposed 3D clinostats with improved control schemes and mechanical configurations [15,28]. For instance, Kim et al. [28] introduced an algorithm in which the angular velocity of one frame is fixed while the other is varied, aiming to optimize the simulated µg effect on biological samples. Recent studies have also reported compact 3D clinostats fabricated by means of 3D printing methods, offering increased accessibility and customization [15,29].

Similarly, several RPMs designs were proposed in literature. Wuest et al. [30] developed an RPM integrating an incubator within the inner frame to maintain controlled culture conditions, using an algorithm where both frames rotate at constant velocity, but the rotation direction is inverted at randomly chosen time points. Wubshet et al. [31] designed a compact RPM compatible with standard tissue culture incubators and implemented a control algorithm that ensures equal probability of all spatial orientations. Yotov et al. [17] extended RPM capabilities by enabling simultaneous use with up to four experimental animals, facilitating in vivo studies.

Despite its well-established use in the scientific community, biological studies employing clinostats or RPMs generally did not provide a detailed description of the hardware or algorithms used to generate simulated µg (s-µg). Such details are crucial as they can substantially influence residual gravity levels and, consequently, biological outcomes.

A rare contribution to the description of the RPMs possible uses was provided by Kim and colleagues [24] who investigated the influence of different time-varying angular velocity profiles for the RPM frames in simulating micro- and hypo-gravity conditions. While the altered gravity conditions are well simulated, the authors noted that further studies are required to investigate the effect of the sudden angular velocity changes on the biological sample.

In this context, we aimed to provide a quantitative characterization of a µg simulator capable of operating in different configurations, namely 2D and 3D clinostat modes, and RPM mode. Specifically, we investigated how these operating modes influence the resulting time-averaged gravity levels, and centrifugal accelerations experienced in the RPM inner frame. To this end, experimental measurements of angular velocity obtained using magneto-inertial measurement units (MIMUs) were integrated with a computational model to simulate rotational dynamics and predict the resulting gravitational environment. Finally, the biological impact of the different operating configurations was evaluated by analysing the behaviour of a neuronal cell model exposed to each condition. This combined mechanical-computational-biological approach aims to provide a quantitative framework for assessing RPM performance and supporting reproducibility in ground-based microgravity studies.

## Methods

### Experimental measurements

In this study, experimental data was acquired on the system for µg simulation RPM 2.0 (Yuri Gravity, Meckenbeuren, Germany).

To characterize the rotational behaviour, wireless MIMUs (Shimmer, Dublin, Ireland) were placed on both RPM frames. The MIMU’s 3D angular velocity was collected via Bluetooth at 100.21 Hz in packets of a nominal size of 0.2 s (20 samples) using MATLAB R2024a (The MathWorks Inc., Natick, MA, USA).

**Fig 1:**
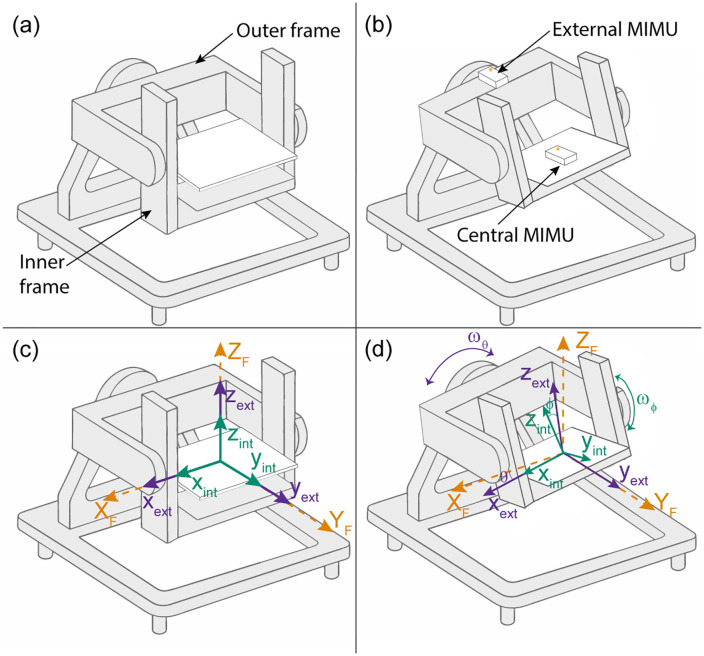
Random Positioning Machine set up and coordinate systems. (a) Schematic representation of the Random Positioning Machine (RPM) used in the study. (b) Schematic representation of the RPM set up with MIMU sensors located in the centre of the inner frame and midway along the length of the outer frame. (c) and (d) illustrate a schematic representation of the RPM coordinate systems of the outer frame (x_ext_,y_ext_,z_ext_) and inner frame (x_int_, y_int_, z_int_) in initial aligned position and instantaneous position during rotation, respectively. Dashed orange arrows represent the fixed coordinate system (X_F_,Y_F_, Z_F_). ω_θ_ and ω_ϕ_ are the angular velocities of the outer and inner frames, respectively.

MIMUs were positioned at two locations on the RPM structure: (i) in the centre of the inner frame (referred as “centred position”); (ii) midway along the length of the outer frame (referred as “external frame position”) (Fig 1b).

The RPM was operated in three different modes:

- **Mode A**: rotation occurs around the two axes with randomly changing rotational speed and directions. Both frames operate at a maximum angular velocity of 60°/s (absolute value) each. The minimum angular velocity was selected to 80% of the maximum angular velocity, i.e., 48°/s, in accordance with the manufacturer’s recommendations. Due to the stochastic nature of this operating mode, five recordings of 10 min each were performed.
- **Mode B**: rotation occurs around the two axes with unidirectional rotation. Both frames operate at a maximum angular velocity of 60°/s (absolute value) each. The minimum angular velocity was selected to 80% of the maximum angular velocity, i.e., 48°/s, as suggested by the manufacturer.
- **Mode C**: inner frame rotates at constant velocity (60°/s) and direction.

At the beginning of the recording session, a preliminary measurement was performed consisting of a one-minute static MIMUs acquisition to estimate the gyroscope bias. This bias was subsequently subtracted from the angular velocity signals recorded during the experiments.

All data processing and analysis were performed in MATLAB R2024a. The operating mode A is characterized by different parameters that contribute to achieve time averaged values of μg, including the angular velocities of the RPM rotating frames, and the number of changes of rotation direction during a time interval. Based on the Bonn criteria proposed by Hammond et al. [23] for reporting RPM experiments, the angular velocity signals were analysed to extract the following parameters describing the device rotation behaviour: (i) number of angular velocity zero crossings, (ii) time interval between two zero crossings of the angular velocity. Average values of these parameters achieved over the five recordings (of 10 min each) from the centred position and external MIMU are reported in Table 1.

**Table 1:** Operating parameters that characterize the trajectory developed during the RPM working mode A obtained from the post processing of MIMUs.

	Central MIMU			External MIMU		
	Mean	Min	Max	Mean	Min	Max
Number of zero crossings	10	5	14	13	12	14
Time interval between two successive zero crossings	25.49 s	5.67 s	115.92 s	21.05 s	5.16 s	78.51 s

The experimentally derived parameters were subsequently used to define the stochastic angular velocity profiles implemented in the computational model, allowing the computer simulations to reproduce realistic motion characteristics of the RPM.

### Computer simulations

A computational model that simulates the trajectory of the acceleration unit vector during RPM rotation was developed. To investigate the influence of operational modes on the development of conditions of simulated µg, we considered different working conditions:

- **RPM Mode A**: rotation occurs around the two RPM axes changing rotational speed and direction randomly.
- **RPM Mode B**: rotation occurs around the two RPM axes at a constant angular velocity for both frames.
- **Clinostat mode A**: inner frame randomly changes angular velocity value and direction. The outer frame remains still.
- **Clinostat mode B**: inner frame rotates at a constant angular velocity.

Descriptions of the algorithms implemented for simulating the different working conditions are provided below.

#### RPM mode A.

This mode simulates independent rotations of the RPM frames by randomly varying angular velocities and directions. Parameters obtained from the experimental data (Table 1) were used in the development of the stochastic angular velocity profile implemented in the mathematical model, allowing the simulations to mimic realistic motion profiles. In particular, the time points at which the rotation direction was inverted were randomly generated, but constrained by the experimentally observed time intervals between consecutive zero crossings of the angular velocity. Velocity transitions between forward and backward rotation were modelled as smooth variations governed by a randomly generated angular acceleration (mean 15°/s^2^, standard deviation 10°/s^2^). The latter values were derived directly from the MIMU data and are consistent with previously reported RPM operating conditions [32].

A fixed coordinate system CS_F_ with the origin O_F_ located at the rotational centre of the RPM was defined, the Y_F_ axis lying along the outer frame rotational axis, and the Z_F_ axis oriented along the direction of gravity (Fig 1c). The RPM motion can be mathematically expressed by two coordinate systems attached to the outer, CS_ext_ (x_ext_,y_ext_,z_ext_), and inner, CS_int_ (x_int_, y_int_, z_int_), frames respectively (Fig 1c). The gravity vector is expressed in the CS_int_ by employing two rotational matrices as follows [30]:


$$\begin{array}{c} \vec{g_{int}}=\overset{-\text{1}}{\underset{X}{R}}\cdot \overset{-\text{1}}{\underset{Y}{R}}\cdot \vec{g_{G}} \end{array}$$ (1)

where


$$\begin{array}{c} R_{X}=[\begin{array}{ccc} \text{1} & \text{0} & \text{0}\\ \text{0} & \text{cos}(\phi ) & -sin(\phi )\\ \text{0} & sin(\phi ) & cos(\phi ) \end{array}] \end{array}$$ (2)

$$\begin{array}{c} R_{Y}=[\begin{array}{ccc} \text{cos}(\theta ) & \text{0} & sin(\theta )\\ \text{0} & \text{1} & \text{0}\\ -sin(\theta ) & \text{0} & cos(\theta ) \end{array}] \end{array}$$ (3)

$$\begin{array}{c} \vec{g_{F}}=[\begin{array}{c} \text{0}\\ \text{0}\\ -\text{1} \end{array}] \end{array}$$ (4)

And thus:


$$\begin{array}{c} \vec{g_{int}}=[\begin{array}{c} -\text{sin}(\theta )\\ \text{cos}\left(\theta )\cdot \text{sin}(\phi \right)\\ -\text{cos}\left(\theta )\cdot \text{cos}(\phi \right) \end{array}] \end{array}$$ (5)

The model generated the acceleration vector expressed in CS_int_ at discrete time points, i.e., Δt = 0.01 s. The trajectory is composed of 1.8 ∙ 10^^6^^ points corresponding to a 5-hour long simulated experiment.

We considered the initial vector (P_0_ = [0, 0, −1]^^T^^) and chose an initial direction. Its trajectory was continuously updated by applying sequential rotations. Thus, the next position of the vector tip considers a rotation of the outer frame around y_ext_ axis by an angle θ achieved as $\theta =\;\omega_{\theta }(t)\cdot \Delta t$ and a rotation of the inner frame around x_int_ axis by an angle ϕ achieved as $\phi {=\;\omega }_{\phi }(t)\cdot \Delta t$, where Δt = 0.01 s, ω_θ_(t) and ω_ϕ_(t) are the angular velocities of the outer and inner frames, respectively.

Rotational velocity of RPM is proven influencing µg simulation [24,30]. To investigate the influence of the angular velocity magnitude, simulations were repeated across a range of maximum velocities from 30°/s to 120°/s at steps of 10°/s. As it occurs in the experimental RPM rotation, the same value of maximum angular velocity is set for both inner and outer frames. For each case, the minimum angular velocity is set to be 80% of the maximum angular velocity. Rotational velocity in the range 30°/s – 120°/s is typically utilized for studying cells [33] or plants [19].

For each maximum angular velocity considered, 500 iterations of 5 hours have been performed.

As the RPM rotated, the acceleration magnitude in the CS_*int*_ varied continuously from -1g to 1g, with g = 9.81m/s^^2^^. The mean value of the accumulated acceleration was calculated using the following equation (Eq. 6) [30]:


$$\begin{array}{c} G_{mean}=\sqrt{{\left(G_{Xmean}\right)}^{\text{2}}+{\left(G_{Ymean}\right)}^{\text{2}}+{\left(G_{Zmean}\right)}^{\text{2}}} \end{array}$$ (6)

$$\begin{array}{c} G_{Xmean}=\frac{\text{1}}{n}\sum_{\text{1}}^{n}g_{x} \end{array}$$ (7)

$$\begin{array}{c} G_{Ymean}=\frac{\text{1}}{n}\sum_{\text{1}}^{n}g_{y} \end{array}$$ (8)

$$\begin{array}{c} G_{Zmean}=\frac{\text{1}}{n}\sum_{\text{1}}^{n}g_{z} \end{array}$$ (9)

where g_x_, g_y_ and g_z_ are the acceleration values of the three axes, n is the total number of measured accelerations, G_Xmean_, G_Ymean_, and G_Zmean_ are the mean acceleration values of the three axes, and G_mean_ is the sum of the mean acceleration values of each axis.

The centrifugal acceleration was also computed as [11]:


$$\begin{array}{c} a_{centrifugal}=\overset{\text{2}}{\underset{int}{\omega }}\cdot d \end{array}$$ (10)

where ω_int_ is the angular velocity calculated in the inner frame and d is the distance from the centre of rotation.

#### RPM mode B.

In this configuration, unidirectional rotation and constant angular velocity are considered for both frames. Simulations were performed by varying the value of the angular velocity from 30°/s to 120°/s with increments of 10°/s in order to analyse its influence on the time averaged gravity. Both frames are assumed to rotate at the same angular velocity. For each maximum value of angular velocity, one iteration was performed, simulating an experiment of 5 hours.

The gravity vector trajectory, the time averaged gravity and the centrifugal acceleration are calculated as described for the RPM mode A.

#### Clinostat mode A.

This configuration simulates a 2D clinostat where only the inner frame rotates with randomly varying angular velocity and direction, while the outer frame remains still. Angular velocities ranged from 30°/s to 120°/s (in 10°/s increments). For each case, the minimum angular velocity is set to be 80% of the maximum angular velocity considered. The angular velocity profile was generated using the same algorithm described in RPM Mode A. For each maximum value of angular velocity, 500 iterations were performed, each simulating an experiment of 5 hours.

The gravity vector is expressed in the local sample frame (CS_int_) as follows:


$$\begin{array}{c} \vec{g_{int}}=\overset{-\text{1}}{\underset{X}{R}}\cdot \vec{g_{F}}=[\begin{array}{c} \text{0}\\ -\text{sin}(\phi )\\ -\text{cos}(\phi ) \end{array}] \end{array}$$ (11)

The time averaged gravity and the centrifugal acceleration are calculated as described for the RPM mode A.

#### Clinostat mode B.

In this operating mode, the inner frame is characterized by unidirectional rotation and constant angular velocity, while the outer frame remains fixed. Values of angular velocity spanning from 30°/s to 120°/s at steps of 10°/s were considered. For each maximum value of angular velocity, one iteration was performed, simulating an experiment of 5 hours. The gravity vector trajectory, the time averaged gravity and the centrifugal acceleration are calculated as described for the Clinostat mode A.

### Cell culture and s-μg based experiments

SH-SY5Y cells (ATCC number: CRL-2266) were cultured in DMEM/F-12, GlutaMAX™ medium (10565018; Gibco) with 10% fetal bovine serum (FBS, A5256701, ThermoFisher) and 1x Penicillin/Streptomycin (Pen/Strep, 15070063, ThermoFisher) at 37° C and 5% CO_2_.

The culture flasks containing the cells were completely filled with medium to minimize shear stress and allow the cells to remain in a state of free-fall. This setup also prevents the formation of air bubbles during RPM rotation, which is crucial for maintaining consistent s-µg conditions. The control group was subjected to the same environmental parameters; specifically, the control flasks were placed on the static platform of the RPM so that the cells experienced identical mechanical vibrations without exposure to s-µg. The control flasks were filled with the same volume of medium as those under s-µg conditions.

Upon seeding, cells were allowed to attach to the flasks surface for 24 hours. Cells were then moved to s-µg or 1g positions, and incubated for 24 hours. Three independent experiments were performed using the RPM operated in Mode A, Mode B, and Mode C, respectively, to evaluate the biological effects associated with each operating configuration.

Samples were then used for downstream analysis

### Immunostaining and Confocal imaging

Immunostaining was performed as previously described [34]. The primary antibody was added at the following concentration: mouse anti a-syn antibody 1:750 (SIG-39730; BioLegend). The secondary antibody was added at the following concentrations: Goat anti-Mouse IgG (H + L) Highly Cross-Adsorbed Secondary Antibody, Alexa Fluor™ Plus 488 (A32723; Invitrogen). 1ng/ml of DAPI solution (62249; Thermo Scientific) was used to visualize the nuclei. Images were acquired using a Leica K3C and analysed using 3D Thunder Analysis.

#### Data analysis.

Statistical analyses were performed using GraphPad Prism version 9.1. Data distribution was first assessed with the Shapiro–Wilk test to determine normality. For non-normally distributed data, group differences were evaluated using the Mann-Whitney test. For normally distributed data with three or more groups, differences were analysed using t-test. Each independent experiment was repeated three times. Data are presented as mean ± standard error of the mean (SEM), and p values < 0.05 were considered statistically significant.

## Results

### Mechanical characterization of RPM operating modes

**Fig 2:**
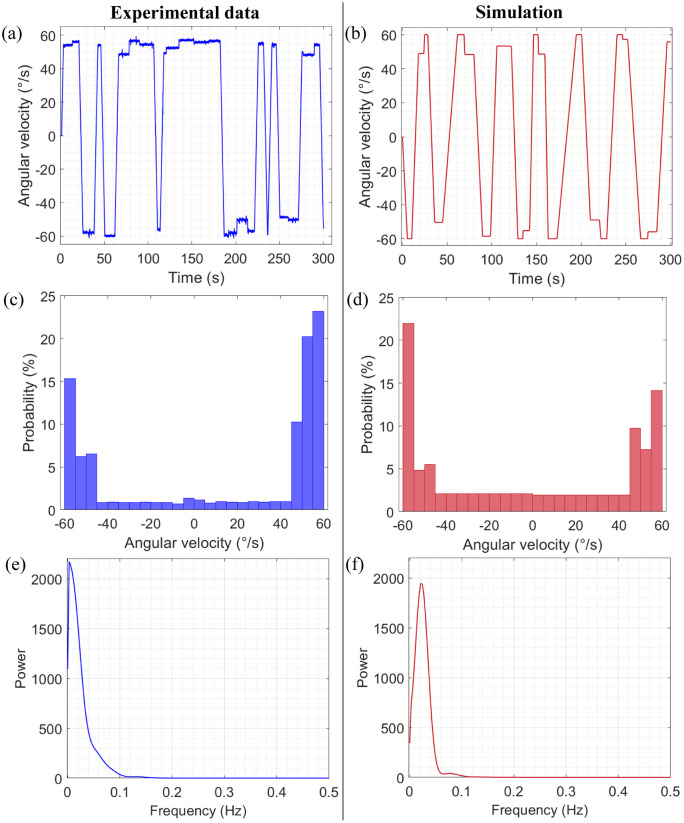
Typical example of angular velocity recorded during RPM operation. (a) Angular velocity measured by MIMU placed on the centre of the RPM platform. (b) Angular velocity developed by the computational model. (c) Histogram of experimentally measured angular velocity values. (d) Histogram of simulated angular velocity values. Periodogram of measured (e) and simulated (f) angular velocity. The example corresponds to the Mode A configuration and highlights the ability of the computational model to reproduce both the temporal dynamics and the statistical distribution of the experimentally observed RPM motion.

To assess the performance of the RPM under various operational modes, a mathematical model was developed to simulate the dispersion of the gravity vector. Angular velocity profiles used in the model were derived from the experimental data acquired by means of MIMU sensors. Fig 2a displays a typical time-varying angular velocity profile recorded by the MIMU placed at the centre of the RPM inner frame under RPM Mode A working conditions. As shown, the angular velocity varies in both magnitude and direction, consistent with the operating mode. The corresponding histogram (Fig 2c) reveals a bimodal distribution of the angular velocity magnitudes. The peaks are reported for values of velocity in the two ranges [55°/s; 60°/s] and [−55°/s; −60°/s] in agreement with the selected operating mode. The computer simulation model reproduced similar behaviour with respect to the experimental data, both in terms of time-domain signals (Fig 2b) and statistical distribution (Fig 2d). Furthermore, the periodograms in Fig 2e and 2f confirm a comparable spectral content between measured and simulated angular velocity signals.

**Fig 3:**
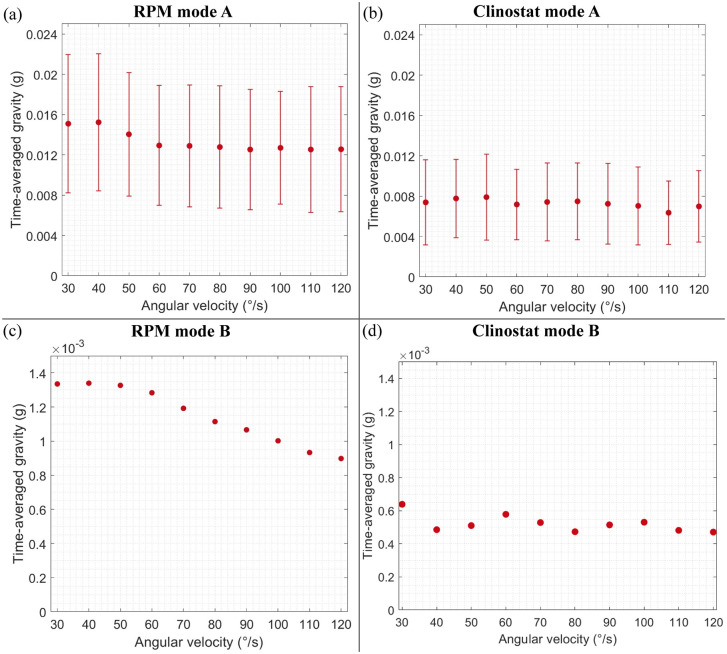
Mean of the time-averaged gravity achieved from the mathematical model as a function of angular velocity for the different working modes. (a) RPM Mode A, (b) Clinostat Mode A, (c) RPM Mode B, (d) Clinostat Mode B. Each data point in plots (a) and (b) represents the average of 500 simulation iterations, with error bars indicating standard deviation. Plots (c) and (d) represent single deterministic simulations per angular velocity.

Fig 3 presents the mean of the time-averaged gravity computed across the angular velocity range (30°/s to 120°/s) using the mathematical model. For the randomized operating modes, i.e., RPM Mode A and Clinostat Mode A (Fig 3a, b), each data point represents the mean of 500 simulation iterations per velocity value. In RPM Mode A, a decreasing trend in the time-averaged gravity is achieved for higher angular velocity values. In fact, for velocities in the range 30°/s – 50°/s, the time-averaged gravity is approximately 0.016 g, whereas for higher velocities, it diminishes around 0.012 g. However, across all angular velocities, the resulting average gravity remains in the 10^^−2^^ g range, which is consistent with values commonly used in experiments with μg simulators [19]. For Clinostat Mode A, nearly the same value of time-averaged gravity is achieved for all angular velocities, i.e., 0.008 g, showing also minor variability in comparison with the RPM Mode A.

For RPM Mode B and Clinostat Mode B (Fig 3c, d), which are characterized by unidirectional rotation, only a single iteration was performed per velocity value due to their deterministic nature. These operating modes are characterized by lower values of time-averaged gravity with respect to the randomized modes. Specifically, in Random Mode B a decreasing trend of time-averaged gravity is observed from 1.4 ·10^^-3^^ g at 30°/s to approximately 0.9 ·10^^-3^^ g for higher values of velocity. Clinostat Mode B consistently yielded the lowest values, around 0.5 ·10^^-3^^ g, with minimal variation across the tested velocities.

**Fig 4:**
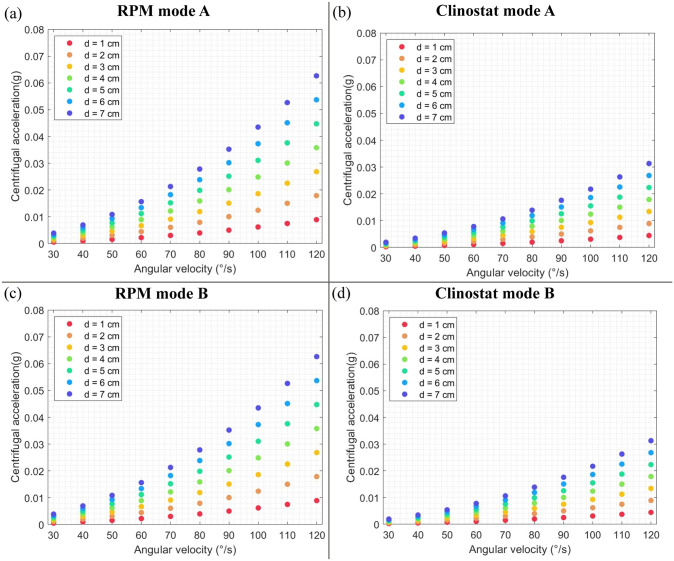
Centrifugal acceleration achieved from the mathematical model as a function of angular velocity and radial distance (d = 1–7 cm) for each simulated working mode. (a) RPM Mode A, (b) Clinostat Mode A, (c) RPM Mode B, (d) Clinostat Mode B.

Finally, the maximum centrifugal acceleration experienced under each operating condition is presented in Fig 4. For the randomized working modes (Fig 4a, b), the plotted values represent the mean of the maximum centrifugal acceleration observed across the 500 simulations for each combination of angular velocity and radial distance, i.e., d ϵ [1 cm; 7 cm]. For deterministic modes (Fig 4c, d), results correspond to the single maximum value obtained per velocity-distance pair. As expected, centrifugal acceleration increases with angular velocity and with the distance from the centre of rotation. This trend is consistent across all working modes. In addition, RPM modes generally exhibit higher values of centrifugal acceleration with respect to the clinostat modes. These values, however, are two orders of magnitude lower than the acceleration of gravity.

### Effects of different RPM set-ups on neuronal cell line physiology

**Fig 5:**
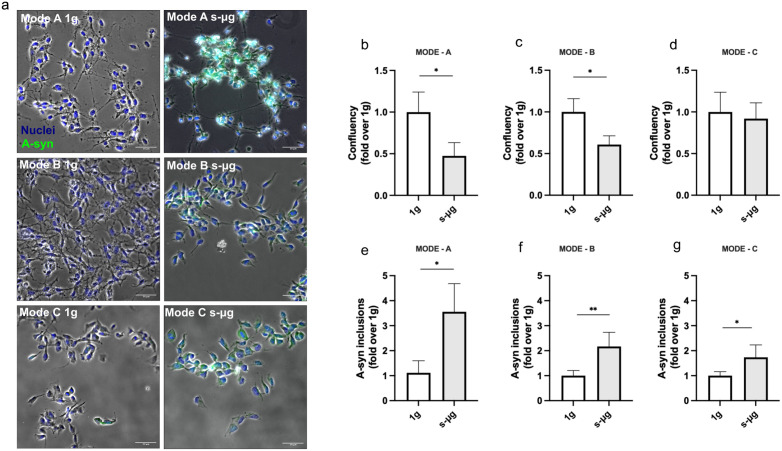
Effects of different RPM settings on neuronal cell model physiology. a) representative images of SH-SY5Y culture in 1g or s-μg conditions using different RPM settings. b) quantification confluency using RPM Mode A. c) quantification confluency using RPM Mode B. d) quantification confluency using RPM Mode C. e) quantification a-syn inclusions using RPM Mode A. f) quantification a-syn inclusions using RPM Mode B. g) quantification a-syn inclusions using RPM Mode C. Data are presented as mean±SEM of 3 independent experiments with a minimum of 3 technical replicates. * = P < 0.05; ** = P < 0.01.

The RPM offers multiple options to achieve s-μg. To study the effects of different RPM parameters on cellular physiology we employed a neuronal cell line model, SH-SY5Y, and exposed it to s-μg using different RPM settings. We first focused on confluency, as this parameter is a routinely used readout for cellular vitality and proliferation [35] and measured it using brightfield microscopy (Fig 5a). Our analysis revealed that both Modes A and B resulted in an approximately 50% reduction in cellular confluency (Fig 5b, c). Cells exposed to s-µg using Mode C also showed reduced confluency, but to a lesser extent (~20%) (Fig 5d).

To further characterize the effects of different RPM settings on cellular processes, particularly protein aggregation, we decided to investigate the levels of alpha-synuclein (a-syn) inclusions upon exposure to s-µg. Confocal microscopy combined with AI-based image analysis revealed increased levels of a-syn inclusions in both Modes A and B (Fig 5e, f). Mode C generated s-µg also produced an increase in a-syn inclusions, but to a more limited extent (Fig 5g). Our findings demonstrate that different RPM set-ups have, overall, a similar impact on the cellular physiology of SH-SY5Y cells.

## Discussion

Although space provides the most authentic environment for investigating the effects of µg on biological systems, access to spaceflight opportunities is extremely limited due to high costs, logistical constraints, and restricted experimental capacity. As an alternative, ground-based µg simulators, such as clinostats and RPM, are widely used to replicate the effects of weightlessness on Earth [12].

These simulators operate by continuously altering the orientation of a biological sample relative to the Earth’s gravity vector. When the reorientation occurs faster than the system’s biological response time, the time-averaged effect of gravity can approximate that of true µg [10]. The scientific community has largely accepted that ground-based simulators can reproduce key features of µg [22]. However, comparative evaluations of different working modes of these simulators and thorough assessments of mechanical and biological side effects remain essential [18,36].

In this context, our study aimed to (i) provide a mechanical characterization of different RPM working modes and (ii) assess whether these modes influence the simulation of µg conditions. Specifically, we analysed randomized and unidirectional RPM working configurations, along with single-axis clinostat modes, focusing on their impact on gravity vector dispersion and potential implications for biological experiments.

The functioning of the RPM was first verified by positioning two MIMUs on the inner and outer frame respectively. RPM Mode A, which operates with randomly varying speed and direction, displayed a bimodal distribution of angular velocity (Fig 2a, c, e), consistent with system expectations. This behaviour was accurately reproduced by the computational model (Fig 2b, d, f), confirming its reliability in simulating realistic RPM motion.

Overall, the predictive model highlights that all operating modes represent an appropriate setting to simulate µg. All working modes maintained a mean residual gravity aligned with values observed in low Earth orbit and other studies based on µg simulators [25,30]. As shown in Fig 3, RPM Mode A exhibited a decreasing trend in mean gravity with increasing angular velocity, ranging from approximately 0.016 g at 30–50°/s to 0.012 g at higher speeds. Clinostat Mode A, in contrast, maintained consistent values near 0.008 g with lower variability. The unidirectional modes, RPM Mode B and Clinostat Mode B, showed even lower residual gravity, in the range of 10 ^−3^ g, with Clinostat Mode B reaching the lowest values (~ 0.5· 10^^-3^^ g). These results indicate that constant angular motion, while more predictable, can be mechanically effective in reducing net gravity, though potentially at the expense of biological realism.

The residual gravity levels obtained in this study are consistent with values typically reported for ground-based microgravity simulators. Previous studies using RPM or clinostat platforms generally report time-averaged gravity values in the range of 10^-2^–10^^-3^^ g depending on the rotation profile and experimental configuration [15,24,28,30]. The values obtained with the randomized RPM configuration in the present study (~10^^-2^^ g) fall within this commonly reported range, while the unidirectional rotating modes reached lower values (~10^^-3^^ g), comparable with those observed in some clinostat configuration [37].

Centrifugal acceleration (Fig 4) increased with both angular velocity and distance from the rotational centre. RPM configurations generated higher peak values than clinostat modes. However, these accelerations remained below 0.08 g, in agreement with other studies available in literature [30].

The findings provide key insights into how different rotation profiles may influence biological responses. The magnitude of residual gravity and centrifugal forces contribute to the cell’s mechanotransduction environment. Clinostat modes could be preferred when minimizing shear and mechanical stress, but their predictable motion may allow cells to adapt, potentially weakening the µg effect over time. In contrast, the non-repetitive and randomized motion of RPM modes prevents directional adaptation, simulating a more dynamic and sustained µg -like environment [27]. However, these advantages must be balanced against increased internal vibration and acceleration fluctuations, which may introduce additional biological effects.

The literature confirms that cellular responses to simulated µg are highly cell-type dependent. For example, Brungs et al. [36] observed that immune cells reduced reactive oxygen species production under clinorotation, but increased it under RPM exposure, concluding that RPM may not be suitable for all systems in its random mode. Hauslage et al. [18] reported higher mechanical stress levels on P. noctiluca cells in an RPM compared to a clinostat. Conversely, other studies support the effectiveness of RPM. Kraft et al. [21] demonstrated that RPM simulates weightlessness well for Arabidopsis thaliana root cells. Eaton et al. [38] showed that 3D RPM motion had a stronger impact than 2D modes on zebrafish larvae.

The present investigation considers SH-SY5Y cells, which are routinely used as a model for studying neurodegenerative disorders [34,39]. This line presents cancerous behaviour, similar to other cancer lines, whilst recapitulating some neuronal features, such as the expression of tyrosine hydroxylase. Our findings in SH-SY5Y cells demonstrated a similar response across all RPM modes, with variations observed only in confluency measurement when using clinostat mode, likely due to the significantly lower level of s-µg achieved. In addition to this, the clinostat mode exploits a single axis rotation, which may reduce the shear forces experienced by cells and therefore affect their capacity to remain attached to the flask [40].

Consistent with our previous work, which showed that SH-SY5Y cells cultured under s-µg conditions exhibit increased levels of α-syn inclusions [41], we observed an increase in misfolded α-syn across the different RPM modes. These results confirm that the observed effects are driven by exposure of the neuronal cell line to simulated µg rather than by the specific RPM configuration.

These results highlight the importance of carefully considering the characteristics of µg simulation platforms and their operational parameters when interpreting biological outcomes, rather than implying that µg levels can be adapted to specific cell types. In real µg environments, such as on the International Space Station, biological systems are exposed to a uniform µg condition, independent of the biological model under investigation. Therefore, differences observed across ground based simulators should be attributed to platform-specific features and residual mechanical cues rather than to variations in µg itself.

An important consideration when interpreting our results is the relationship between computed mechanical metrics and observed biological outcomes. While RPM Mode A achieved a time-averaged gravity of approximately 0.012–0.016 g compared to 0.0010–0.0014 g in Mode B, a roughly ten-fold difference, both modes produced nearly identical biological responses in SH-SY5Y cells. This apparent contradiction resolves when considering that both gravity values fall within the physiological range documented in real spacecraft environments [42] and extensively used in published ground-based simulator studies [11, 37]. Biological systems exhibit threshold behaviour for gravity sensing. Once gravitational acceleration is sufficiently reduced to prevent cellular directional adaptation, further reduction produces no additional response, as demonstrated in partial gravity studies where cellular responses plateau between lunar (0.17 g) and Martian (0.38 g) gravity levels [19]. Therefore, the saturation of cellular responses across both RPM modes likely reflects the fact that a biological threshold has been surpassed.

An important limitation of this study should be acknowledged. While the model incorporates realistic angular velocity profiles and effectively simulates gravity vector dispersion, it does not capture all dynamic interactions present within the cell culture environment, such as fluid flow-induced shear stress. This limitation is common to ground-based µg simulators and should be considered when interpreting the results. Nevertheless, simplified order-of-magnitude estimations based on analytical approximations [43] suggested shear stress values in the mPa range across the investigated operating modes consistent with previous RPM studies [26, 43] and substantially lower than the levels typically associated with pronounced shear-induced effects in neuron-like cells [44]. Additionally, the use of undifferentiated SH-SY5Y should be considered when translating these findings to more advanced neuronal models. This line does not present a typical neuronal phenotype characterised by a G0 phase, despite expressing neuronal markers, but presents an active proliferation typical of cancerous lines.

Despite these constraints, the consistency of the cellular responses observed across different RPM configurations strengthens the conclusion that s-µg exposure is the primary driver of the reported biological effects, underscoring the robustness of the experimental design and the relevance of the RPM as a reliable tool for studying cell responses to µg [12,36].

## Conclusion

The RPM is a pivotal instrument for simulating µg, allowing for the investigation of the effects of weightlessness on biological systems within a controlled laboratory setting. Comprehensive mechanical characterization of the RPM is crucial to ensure its reliability and efficacy. The present study highlights that both RPM and clinostat working modes can effectively simulate µg conditions, with varying performance depending on motion characteristics.

The computational model developed here represents a tool for quantitatively comparing operational modes and optimizing protocols prior to biological testing. Ultimately, the choice of operating mode must be matched to the biological model and research question, emphasizing the need for continued comparative studies in this field.
